# A prospective observational study of 56 patients treated with ring fixator after a complex tibial fracture

**DOI:** 10.1007/s11751-017-0275-9

**Published:** 2017-02-10

**Authors:** Rasmus Elsoe, Søren Kold, Peter Larsen, Juozas Petruskevicius

**Affiliations:** 1 Department of Orthopaedic Surgery, Aalborg University Hospital, Aalborg University, 18-22 Hobrovej, 9000 Aalborg, Denmark; 2 Department of Occupational Therapy and Physiotherapy, Aalborg University Hospital, Aalborg University, 18-22 Hobrovej, 9000 Aalborg, Denmark

**Keywords:** Ilizarov, Ring fixator, Complex fracture tibial bone, Plateau fracture, Pilon fracture, Short-term outcome

## Abstract

The objective of this prospective study was to evaluate the patient-reported outcomes for patients with complex tibial fractures treated with a ring fixator. The secondary aim was to analyse the variables affecting patient-reported outcomes and time to union. Fifty-six patients participated in the study. The mean age at the time of fracture was 56.5 years (range 30–86). All fractures united during the study period. The ring fixator was removed at an average of 25.3 weeks (range 9–53). During treatment, the function and QOL increased with time. Compared with an established reference population, the study population showed a significantly worse EQ5D-5L index both throughout the treatment period and 8 weeks after frame removal. 18% of patients reported mild to severe depression 8 weeks after frame removal.

**Electronic supplementary material:**

The online version of this article (doi:10.1007/s11751-017-0275-9) contains supplementary material, which is available to authorized users.

## Introduction

Complex fractures of the tibial bone involving the joint surfaces and multi-fragmented tibia shaft fractures with soft tissue damage are challenging [1–3]. Conservative management is often not feasible and, consequently, most fractures are treated operatively [4, 5].

Surgical management methods include open reduction and internal fixation [6], angle-stable locking plates [7], ring fixators [8] and percutaneous screw fixation [9]. The literature does not favour a single surgical method from objective measures or patient-reported outcomes. There are ongoing discussions concerning the patient-reported QOL throughout the treatment period between the different surgical methods.

The authors prefer the use of ring fixation for the treatment of complex fractures of the tibial bone. The period from surgery to union and removal of the frame is considerable and can vary from 8 to 87 weeks [10–12]. To the authors’ knowledge, no studies have evaluated the patient-reported outcomes during the treatment period. Moreover, no studies have undertaken an analysis of the variables affecting short-term patient-reported outcome and with one study only reporting factors affecting time to union [13].

The primary aim of this study was to report the patient-reported quality of life (HRQOL) from surgery to eight weeks after frame removal in patients with a complex tibial fracture. The secondary aim was to analyse variables affecting patient reported outcomes and time to union.

The hypothesis was that patients would report worse outcome compared with the Danish reference population on EQ5D-5L index score from time of surgery to eight weeks after frame removal following a complex tibial fracture.

## Patients and methods

### Study design

The study design was a prospective follow-up study including all patients treated with a ring fixator after a complex fracture of the tibial bone. The Danish Data Protection Agency (J. nr. 2008-58-0028) approved the study. The main outcome measurement was the EQ5D-5L index [14].

### The Trauma Ilizarov Database (TID)

**Fig. 1:**
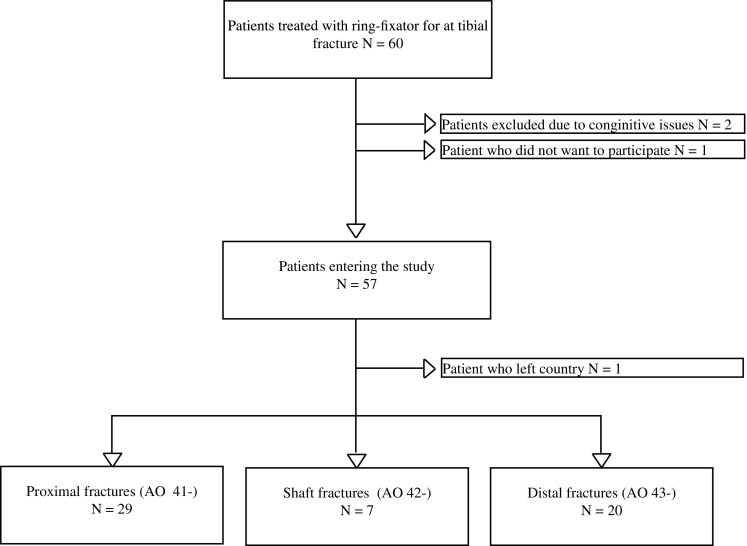
Patient recruitment flow

All patients treated with a ring fixator following a complex fracture of the tibial bone between December 2012 and May 2014 at Aalborg University Hospital, Denmark, were included in the Trauma Ilizarov Database. Patients with complex tibial fractures treated without a ring fixator were excluded. Patients who were unable to fill out the questionnaires due to physical or mental disabilities were excluded. A detailed overview is shown in Fig. 1.

**Fig. 2:**
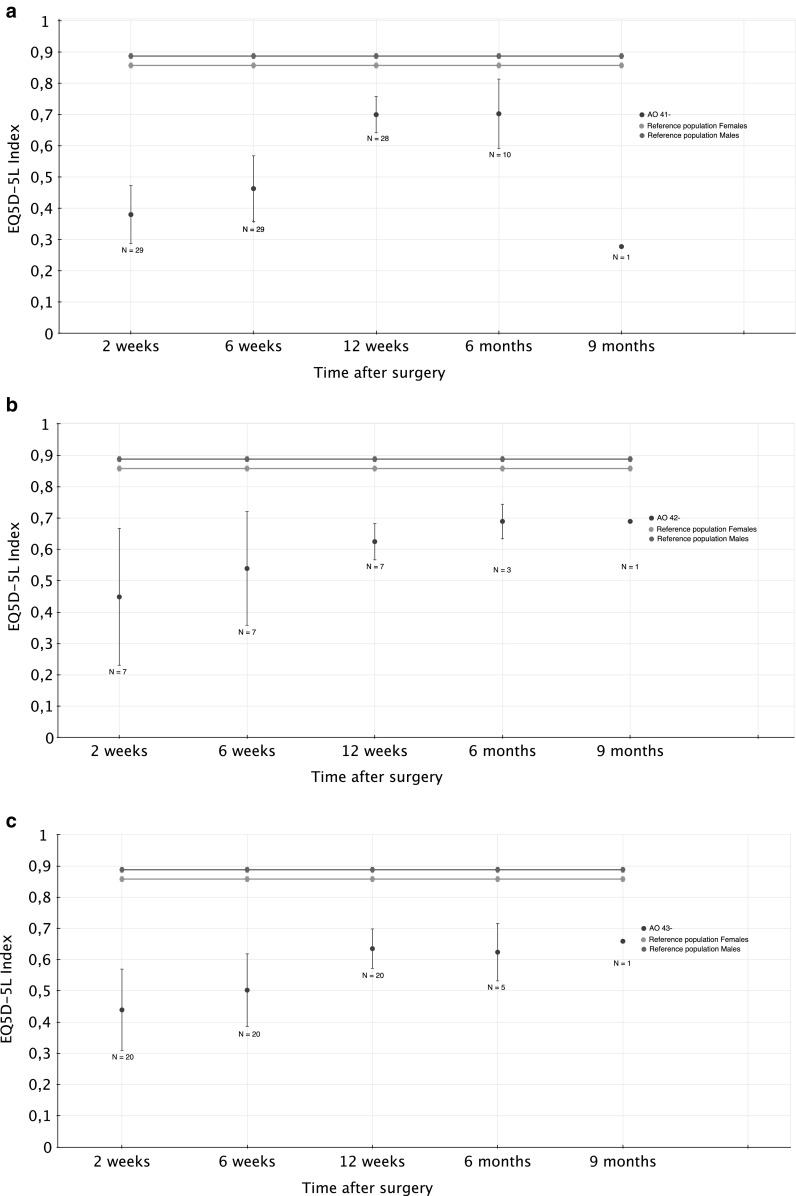
**a** Patient reported outcome, proximal tibial fractures (AO 41-), patient-reported outcome from surgery to frame removal, proximal tibial fractures. **b** Patient reported outcome, tibial shaft fractures (AO 42-), patient-reported outcome from surgery to frame removal, tibial shaft fractures. **c** Patient reported outcome, distal tibial fractures (AO 43-), patient-reported outcome from surgery to frame removal, distal tibial fractures

Patient baseline characteristics were obtained at the time of admission to hospital. All patients were systematically examined at the outpatient clinic after 2, 6 weeks, 3 and 6 months. A final examination was conducted 8 weeks after removal of the fixator.

Data on age, gender, trauma mechanism, type of trauma, fracture classification, type of surgery, comorbidities and complications were registered. Fracture classification was performed using the AO classification [15] and based on a CT scan pre-operatively.

### Surgical treatment

Bicondylar fractures of the tibial bone, complex fractures with soft tissue damage of the tibial shaft and distal fractures of the tibial bone not treatable by intramedullary nailing were all treated by an external ring fixator. The authors preferred to manage proximal and distal tibial fractures with initial screw fixation of joint bearing bone fragments and, if necessary, with exposure of the joint surface. Both autogenous and allogeneous bone grafting were used. The metaphyseal–diaphyseal fractures were bridged by one or more rings. The frame was connected to the bone by hydroxyapatite-coated half-pins and k-wires with olives as needed. After applying the ring fixator alignment was assessed and corrected if needed. Amendments such as footplates and proximal fixation of the femur were used where deemed appropriate.

All patients were systematically examined at the outpatient clinic every 6 weeks until fracture union. In general, patients with fractures of the joint surfaces were kept non-weight bearing for 6 weeks. The decision of fracture union and the removal of the frame was as described by Ramos et al. [8]; the fracture was regarded as united when 3 of 4 cortices on antero-posterior and lateral X-rays showed bridging callus; the fracture was stable under manual stress and the patients were able to walk without pain after the connection rods had been removed.

All patients had a standardized physiotherapy programme from the first day following surgery and daily until discharge. After discharge, the patients were managed in the outpatient clinic. The rehabilitation programme has special focus on knee and ankle range of motion, muscle function and the ability to maintain these functions in conjunction with management of activities of daily living. In general, patients were seen in the outpatient clinic 1–3 times a week for 3–5 months.

### Outcome measurements

#### Patient reported measurements

EQ5D-5L is a standardized and validated instrument to assess health outcome [14]. It consists of 5 dimensions: mobility, self-care, usual activities, pain/discomfort and anxiety/depression and a self-rated health scale on a 20 cm vertical, visual analogue scale with endpoints labelled ‘the best health you can imagine’ and ‘the worst health you can imagine’. Each dimension has 5 levels: no problems, slight problems, moderate problems, severe problems and extreme problems. A Danish data set was used to calculate the EQ5D-5L index [16]. An EQ5D-5L index at 1.0 indicates full health and 0.0 denotes death. Reference population from Denmark is available [17].

Knee Injury and Osteoarthritis Outcome Score (KOOS) [18] is a standardized and validated instrument used to evaluate knees and associated problems. The questionnaire includes 42 items, and each item obtains a score from 0 to 4; a total score from 0 to 100 is calculated for each subscale. A total score of 100 indicates no symptoms and 0 indicates major symptoms. KOOS reference data [19] from a general population-based sample in southern Sweden is available.

The Olerud–Molander Ankle Score (OMAS) [20] is a standardized and validated instrument used to evaluate ankle and associated problems. The OMAS is a patient-reported questionnaire developed to evaluate function after ankle fracture. The scale is a functional rating scale from 0 (totally impaired) to 100 (completely unimpaired) and is based on nine different items: pain, stiffness, swelling, stair climbing, running, jumping, squatting, supports and activities of daily living.

The Major Depression Inventory (MDI) score [21] is a validated system designed to measure depression symptoms in accordance with the symptom guidelines defined by the WHO classification for unipolar depression (ICD-10) and the American Psychiatric Association classification for major depression (DSM-IV). The instrument consists of 12 questions. On a 6-point Likert scale, the individual items measure how much of the time the symptoms have been present during the last 14 days. The MDI was scored according to specific guidelines. A score of 0 indicates no depression and 50 severe depression. The categories, no depression, less than 20, mild, 20–24, moderate, 25–29 and severe depression, 30 or more, were used [21, 22].

#### Radiological outcome measurements

Radiographic examination included X-rays and pre-operative CT scans for all patients. Postoperatively, X-rays of the entire lower leg were obtained and used to evaluate the quality of reduction. Radiological examination was performed at 6 weeks, 3 months and every 6 weeks until union. At the final examination 8 weeks after fixator removal, the radiological assessments were made on AP and lateral X-rays. Proximal tibial fractures were evaluated by alignment and depression of the articular surface and condylar widening as described by Rasmussen et al. [23]. Shaft fractures were evaluated by alignment. Distal fractures were evaluated with regard to alignment, talar subluxation, central depression and mortise widening as described by Ramos et al. [2] Furthermore, an assessment of the postoperative reduction for distal fractures was performed as described by March and co-workers [24], modified by Burwell and Charnley [25]. Two authors carried out radiological evaluations separately (RE & JP). In case of disagreement, consensus was obtained.

#### Objective outcome measurements

##### Range of motion (ROM)

Knee range of motion was assessed by active extension and flexion of the knee with the patient supine on the examination table. The patient was asked to perform maximal flexion and extension, and the angle was measured by a goniometer. Ankle range of motion was assessed by active dorsal and plantar flexion of the talocrural joint with the patient supine on the examination table. The patient was asked to perform maximal dorsal and plantar flexion, and the angle was measured by a goniometer.

Pain was assessed with a visual analogue scale (VAS) ranging from 0 to 100 mm. Patients were asked to classify pain while resting.

#### Statistics

Continuous data were expressed with mean and standard deviation (SD). Categorical data were expressed as frequencies. The assumption of normal distribution variables was checked visually by Q–Q plots. Linear or logistic regression was used to analyse variables affecting time to union and patient-reported outcome. The Chi-squared test was used to compare patients’ reported outcome between categorical variables. A *P* value of <0.05 was considered significant.

The statistical analysis was performed by Stata (version 13).

## Results

A total of 60 patients were treated for a tibial facture with ring fixator during the study period. Four patients met one or more of the exclusion criteria, and 56 patients participated in the study (Fig. 1).

There were 32 females and 24 males in the study population. The mean age at the time of fracture was 56.5 years, range 30–86. The baseline variables for all patients concerning trauma mechanism, type of trauma, fracture classification, open or closed fracture, comorbidities and complications are presented in Table 1. Thirty-two patients (57%) patients had antibiotics during the treatment period due to pin or wire infections. One patient was readmitted to hospital for antibiotics intravenously. Twelve (21%) patients had one or more wires exchanged due to infection. No instances of compartment syndrome or osteomyelitis were observed, and all patients united during the study period.

**Table 1:** Baseline characteristics

Age at time of fracture, mean (range)	56.5 (30–82)
Gender male/female	24/32
Smoker yes/no	37/19
Side of injury, right/left/bilateral	27/27/2
High-/low-energy trauma	19/37
Comorbidities	
ASA score, mean(SD)	1.8 (0.7)
Charlson comobidity score, mean(SD)	2.9 (1.9)
Diabetes mellitus	8
Fracture classification	
AO-41	29
AO-42	7
AO-43	20
Open/closed fracture	9/47
Complications	
Pin site infection, number of patients	33
Pin or wire infection treated in hospital	1
Pin or wire infection treated with peros antibiotics	32
Pin or wire exchange during treatment period	12

Twenty-nine patients presented with a proximal tibia fracture AO 41- (A2 = 1, A3 = 1, C1 = 4, C2 = 1, C3 = 22). Seven patients presented with a complex shaft fracture AO 42- (A1 = 1, A2 = 3, C1 = 2, C3 = 1). Twenty patients presented with a distal fracture AO 43- (A2 = 1, A3 = 4, B1 = 3, B2 = 1, B3 = 3, C1 = 1, C2 = 1, C3 = 6).

### Patient-reported outcome

#### MDI

**Fig. 3:**
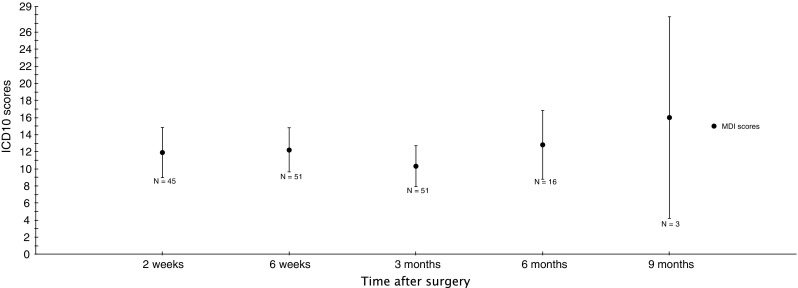
Patient-reported MDI scores from surgery to frame removal

Overall, 18% of patients reported mild to severe depression 8 weeks after frame removal. Five patients reported MDI scores between 20 and 30 indicating mild to moderate depression, and 5 patients had a score of >30 indicating severe depression. No significant difference in MDI scores was observed throughout the treatment period (Fig. 3).

Six patients with proximal fractures, 2 patients with shaft fractures and 2 patients with distal fractures reported mild to severe depression.

#### Proximal fractures (AO 41-)

The mean EQ5D-5L index from surgery to union is presented in Fig. 2. Eight weeks after frame removal, the mean EQ5D-5L index was 0.695 (CI 0.63–0.76). The mean EQ5D-5L VAS was 74.5 (CI 65.2–83.9). Compared with the established reference population from Denmark [17], the study population showed a significantly worse EQ5D-5L index at the time of union (Table 2).

**Table 2:** Patient-reported outcome 8 weeks after frame removal compared with reference populations

	KOOS									
	PAIN		ADL		SYMP		QOL		SPORT	
	Mean	95% CI	Mean	95% CI	Mean	95% CI	Mean	95% CI	Mean	95% CI
Proximal fracture (AO 41-)										
Study population	65.6	56.1–75.2*	69.8	58.6–81.0*	54.5	44.3–64.6*	48	38.1–57.8*	8.6	17.3–39.8*
Reference population**,***		86.7–88.2		86.5–88.1		85.4–86.9		77.4–79.6		72.5–75.1

	EQ5D**-**5L			
	Index		VAS	
	Mean	95% CI	Mean	95% CI
Study population	0.695	0.627–0.763*	74.5	65.2–83.9
Reference population**** (male/female 50–59 years)	0.888/0.858	0.880–0.896/0.850–0.866		
Shaft fracture (AO 42-)				
Study population	0.579	0.429–0.728*	57.9	29.6–86.1
Reference population**** (male/female 50–59 years)	0.888/0.858	0.880–0.896/0.850–0.866		
Distal fracture (AO 43-)				
Study population	0.646	0.570–0.7*	66	55.4–76.5
Reference population**** (male/female 50–59 years)	0.888/0.858	0.880–0.896/0.850–0.866		

* Significantly different compared with reference population

** Paradowski PT et al. BMC Musculoskeletal Disord, 2006^18^

*** Unpublished data. Ewa Roos ‘Personal communication’ Nov 13, 2 01 2. Paradowski et al. 2006

**** Sorensen J et al. Scand. J. Public Health, 2009^16^

Eight weeks after frame removal, the mean KOOS score was pain 65.6 (CI 56.1–75.2), symptoms 54.5 (CI 44.3–64.6), ADL 69.8 (CI 58.6–81.0), sport 28.6 (CI 17.3–39.8) and QOL 48.0 (CI 38.1–57.8). Compared with the established reference population [19], the study population showed a significantly worse KOOS outcome for all the five subgroups (Table 2).

#### Shaft fractures (AO 42-)

The mean EQ5D-5L index from surgery to union is presented in Fig. 2. Eight weeks after frame removal, the mean EQ5D-5L index was 0.58 (CI 0.43–0.73). The mean EQ5D-5L VAS was 57.9 (CI 29.6–86.1). Compared with the established reference population from Denmark [17], the study population showed a significantly worse EQ5D-5L index at the time of union (Table 2).

#### Distal fractures (AO 43-)

The mean EQ5D-5L index from surgery to union is presented in Fig. 2. Eight weeks after frame removal, the mean EQ5D-5L index was 0.65 (CI 0.57–0.72). The mean EQ5D-5L VAS was 66.0 (CI 55.4–76.5). Compared with the established reference population from Denmark [17], the study population showed a significantly worse EQ5D-5L index (Table 2).

The mean Olerud–Molander Ankle Score 8 weeks after frame removal was 40.3 (CI 29.6–50.9). No reference population was available for the Olerud–Molander Ankle Score.

### Radiological outcome measurements

#### Proximal fractures (AO 41-)

All fractures united during the study period. The ring fixator was removed at an average of 23.5 weeks, range 9.1–45.4. At the final examination 8 weeks after frame removal, 9 patients were out of alignment or had an articular depression of more than 3 mm (Table 3).

**Table 3:** Observed deformities, depressions and condylar widening

	Varus deformity measured in °	Valgus deformity measured in °	Flexion deformity measured in °	Extension deformity measured in °	Depression AP mm	Depression lateral mm	Condylar widening mm
Proximal							
Patient ID							
2		5			3		
11			10		2	3	
3			8		1	1	
17					6	3	10
34					6	2	8
39			3		5	3	0
46						4	
52			3		4		
55	4				1		

	Varus deformity measured in °	Valgus deformity measured in °	Flexion deformity measured in °	Extension deformity measured in °
Shaft				
Patient ID				
8				4
13	5			

	Varus deformity measured in °	Valgus deformity measured in °	Flexion deformity measured in °	Extension deformity measured in °	Central depression > 3 mm
Distal					
Patient ID					
5					3
25	3				
26					4
33		3			
45	8				
51					7
53					5

Eight weeks after frame removal, the radiological assessments were made on AP and side X-rays. Proximal tibial fractures were evaluated concerning alignment and depression of the articular surface and condylar widening as described by Rasmussen et al. [22]. Shaft fractures were evaluated concerning alignment. Distal fractures were evaluated with regard to alignment, talar subluxation, central depression and mortise widening as described by Ramos et al. [2] Furthermore, an assessment of the postoperative reduction for distal fractures was performed as described by March and co-workers [23], modified by Burwell & Charnley [24]

#### Shaft fractures (AO 42-)

All fractures united during the study period. The ring fixator was removed at an average of 27.4 weeks, range 16.1–42.0. At the final examination 8 weeks after frame removal, one patient was out of alignment, representing a varus deformity of 5° (Table 3).

#### Distal fractures (AO 43-)

All fractures united during the study period. The ring fixator was removed at an average of 24.9 weeks, range 13.4–51.3. At the final examination 8 weeks after frame removal, three patients were out of alignment and three patients had a central depression of more than 3 mm. No talar subluxation of more than 0.5 mm or mortise widening of more than 0.5 mm was present. The Burwell and Charnley classification shows 12 patients with good reduction, six patients with fair reduction and one with poor reduction (Table 3).

### Objective outcome measurements

#### Proximal fractures (AO 41-)

At the final examination 8 weeks after frame removal, the mean knee flexion was 116.9° (CI 112.1–121.7). Twelve patients experienced a knee extension limitation of 5° or less, and 2 patients had a knee extension limitation exceeding 10°.

The VAS score for rest pain 8 weeks after frame removal was reported with a range from 0 to 6. Twenty-two patients reported no pain, five patients reported VAS between 1 and 5 and two patients reported VAS 6.

#### Shaft fractures (AO 42-)

The VAS score for rest pain 8 weeks after frame removal was reported with a range from 0 to 7. Two patients reported no pain, 4 patients reported VAS between 1 and 5 and 1 patient reported VAS 7.

#### Distal fractures (AO 43-)

At the final examination 8 weeks after frame removal, the mean dorsal flexion of the ankle was 9.5° (CI 5.2–13.7). The mean plantar flexion of the ankle was 22.5° (CI 18.3–26.8).

The VAS score for rest pain 8 weeks after frame removal was reported with a range from 0 to 8. Twelve patients reported no pain, five patients reported VAS between 1 and 5 and two patients reported VAS between 7 and 8.

### Analysis of variables affecting time to union

The analysis of variables affecting time to union shows a significant association between time to union and smoking (*P* = 0.04). No significant association between age, BMI, Charlson comorbidity score, pin or wire infection and high-/low-energy trauma was observed (*P* ≥ 0.05, Table 4).

**Table 4:** Variables affecting time to union and patient-reported outcome

	Time to union	EQ5D-5L
Age	*b* = 0.51, *P* = 0.06	*b* = 0.02, *P* = 0.70
BMI	*b* = 0.24, *P* = 0.63	*b* = 0.06, *P* = 0.56
Smoking	***b*** **=** **0.09,** ***P*** **=** 0.04*	*b* = 0.27, *P* = 0.88
Charlson comorbidity	*b* = 0.07, *P* = 0.05	*b* = 0.007, *P* = 0.32
Pin/wire infection	*b* = 0.07, *P* = 0.11	*b* = 2.13, *P* = 0.26
High-/low-energy trauma	*b* = 0.05, *P* = 0.23	*b* = 0.93, *P* = 0.61

*b* = regression coefficient

Bold represents statistically significant difference

### Analysis of variables affecting patient-reported outcome

Eight weeks after frame removal, baseline variables (age, BMI, Smoking, Charlson comorbidity score, infection and high-/low-energy trauma) show no significant influence on patient-reported outcome (EQ5D-5L; *P* ≥ 0.26, Table 4).

Eight weeks after frame removal, a comparison of patients with a fracture out of alignment or with an articular depression and patients with fractures in alignment or without articular depression shows no significantly worse EQ5D-5L index (*P* = 0.50).

## Discussion

This study shows that ring fixation of complex fractures of the tibial bone has a high rate of union and a low rate of complications. These findings are supported by a number of recent studies [2, 12, 26–28]. Moreover, the fracture and subsequent treatment was associated with significant persisting disability and depression until 8 weeks after removal of the frame.

This is the first study to prospectively evaluate the patient-reported QOL and function throughout the treatment period in patients treated with a ring fixator after a complex tibial bone fracture. Throughout the treatment period, patients with complex fractures of the tibial bone treated with a ring fixator experience worse function and QOL compared with the established reference populations. Unfortunately the study has no information regarding pre-injury health status, and it could be argued that the pre-injury health status of the study population is not comparable to the established national reference population. Skoog et al. [29] have reported comparable pre-injury QOL values in a population of tibial fractures compared to reference populations. The second limitation was the study could not distinguish whether poor QOL was influenced by injury or by the treatment with circular frame.

During the treatment period, function and QOL increased with time. No studies evaluating other surgical treatment methods have prospectively reported the patient-reported QOL from the time of fracture to union. In summary, more research is needed regarding patient-reported function and QOL throughout the treatment period between different surgical methods.

A number of studies have reported the outcome after complex fractures of the tibia bone. Ramos et al. [2, 8] have, in two recent studies, evaluated the patient-reported functional outcome after complex fractures to the distal and proximal end of the tibial bone treated with ring fixator. These studies do not compare the results to an established reference population but still show that, even after successful treatment, patients reported a low score on the KOOS/FAOS subscales for sports and QOL. A retrospective study by Ahearn et al. [28] support these findings and reported poor outcome scores after complex tibial plateau fractures evaluated on WOMAC and SF-36, despite satisfactory reduction and alignment. Furthermore, a large-scale retrospective study by O’Toole et al. [30] reported that the most important drivers in patients’ satisfaction following major lower limb trauma seem to be physical function, less pain, the absence of depression and the ability to return to work. Moreover, O’Toole et al. [30] reported that patients’ satisfaction was not related to details of the injury, patient demographics or psychological profile of the patient. These findings indicate that complex fractures of the tibial bone are severe in nature and may result in some disability. It is the authors’ intention to report the objective and patient-reported outcome 1 and 3 years after frame removal in order to evaluate the development in patient-reported QOL and function.

This study shows an unexpected high rate of mild to severe depression 8 weeks after frame removal. These findings are new and, to the authors’ knowledge, no earlier studies have reported mental disability for the present study population. The severe nature of the fractures and the long treatment period in combination with a high degree of socioeconomic consequences and a significantly worse QOL may be contributory factors leading to mental vulnerability. Krappinger et al. [3] support these findings in a recent study of patients treated with the Ilizarov technique after large post-traumatic tibial bone defects. The study reported a major burden of mental and physical stress for both patients and their relatives. In contrast, Baschera et al. [11] reported no significantly worse SF-12 mental component score compared to a normal population in patients treated with ring fixator after 1–9 years’ follow-up. The overall mental health for patients with complex fractures of the tibial bone may be a point of further interest in clinical evaluation, treatment and research in the future.

This study shows a significant negative effect between smoking and time to union. A recent systematic review by Patel et al. 2013 [31] evaluated the effect of smoking on bone healing after tibial fractures and support the findings from the present study. Patel et al. [31] reported a significant longer time to fracture healing for smokers and concluded an overall negative effect of smoking on bone healing after tibial fractures. In contrast, Alemdaroglu et al. [13] reported no significant difference in the time to union for smokers for patients treated with ring fixator of the tibial bone. This study shows no significant correlation between any of the other baseline characteristics and time to union. The rate of complications in this patient population was low thus larger studies should be conducted to reveal the influence of variables such as high-energy trauma, open fractures, soft tissue injuries, diabetes, age and malnutrition that affect fracture union [13, 32–35].

## Conclusion

This study shows a major morbidity related to the treatment of complex tibial fractures until 8 weeks after frame removal. Treatment of complex tibial fractures involving joint surfaces is challenging, and this study shows a significant burden on QOL, mental and physical disabilities for the patients throughout the prolonged treatment period. Even eight weeks after union and removal of the frame, patients experienced a significantly worse patient-reported outcome compared with an established reference population. At the time of frame removal, no significant difference in EQ5D-5L index between AO type 41-, 42- and 43- was found. Eight weeks after frame removal, 18% of the patients reported mild to severe depression.

## Electronic supplementary material

Below is the link to the electronic supplementary material.

Supplementary material 1 (JPEG 474 kb)

Supplementary material 2 (JPEG 281 kb)

Supplementary material 3 (JPEG 399 kb)
