# Supplementary figures and images for: A prospective observational study of 56 patients treated with ring fixator after a complex tibial fracture
Source: Strategies Trauma Limb Reconstr. 2017 Feb 10;12(1):35–44. doi: 10.1007/s11751-017-0275-9 (PMC5360674; doi:10.1007/s11751-017-0275-9)

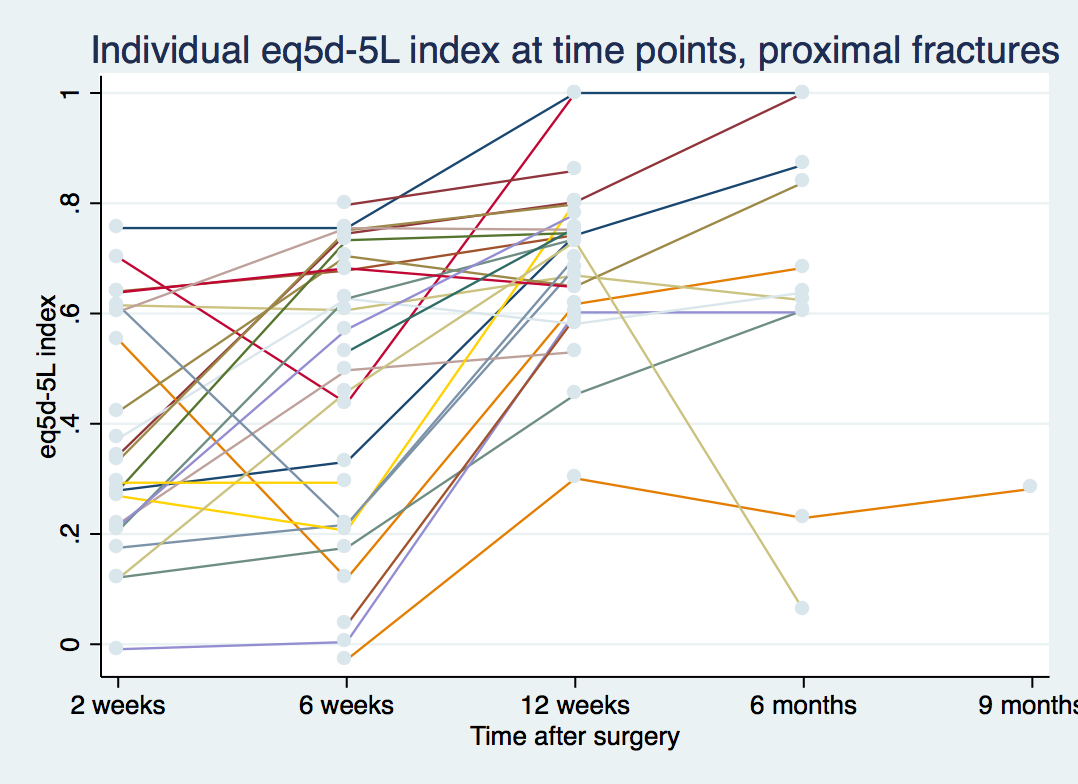

Supplement: Supplementary file 1 — Supplementary material 1 (JPEG 474 kb) [file 11751_2017_275_MOESM1_ESM.jpg]

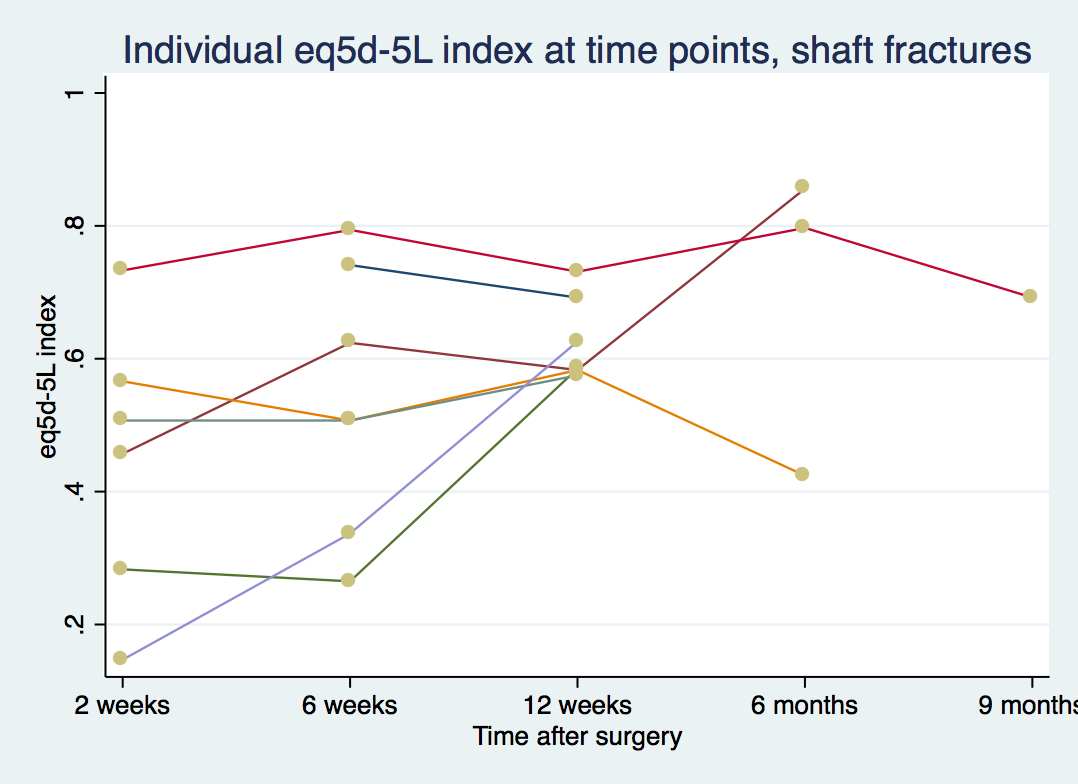

Supplement: Supplementary file 2 — Supplementary material 2 (JPEG 281 kb) [file 11751_2017_275_MOESM2_ESM.jpg]

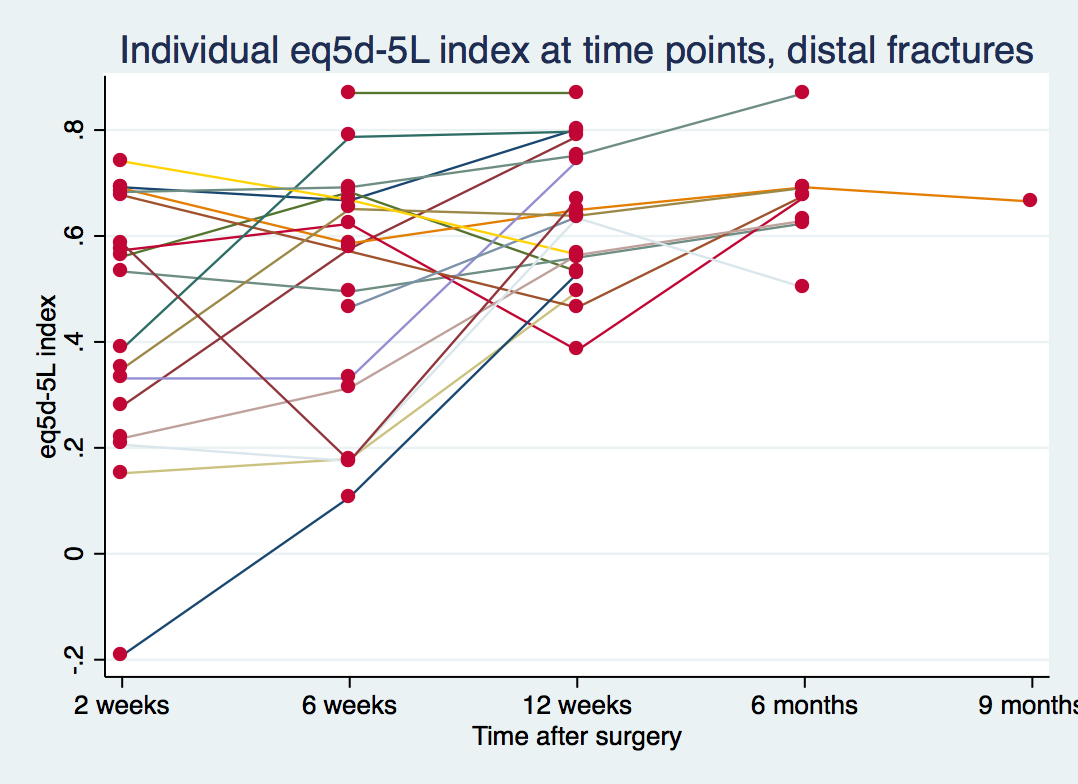

Supplement: Supplementary file 3 — Supplementary material 3 (JPEG 399 kb) [file 11751_2017_275_MOESM3_ESM.jpg]
